# Itch: A Global Problem?

**DOI:** 10.3389/fmed.2021.665575

**Published:** 2021-05-28

**Authors:** Elke Weisshaar

**Affiliations:** Occupational Dermatology, Department of Dermatology, Ruprecht-Karls University Heidelberg, Heidelberg, Germany

**Keywords:** itch, global itch, pruritus, prevalence, prurigo, epidemiology

## Introduction: the Global Burden of Itch

Chronic itch (CI) is defined as itch occurring for 6 weeks and longer (1). Both the frequency and the causes of CI depend on age, predisposition like atopy, underlying diseases, ethnicity, climate/humidity, and especially access to the regional healthcare system (2–5). Different living conditions and rituals in human beings as well as migration also contribute to acute and chronic itch (5, 6). The prevalence of itch appears to differ worldwide (2, 3, 5). Direct comparisons between continents or countries are unusual and rare. There are only a few epidemiological studies on itch outside Europe and North America (2, 3, 5). This may be mainly caused by the facts that there is a lack of dermatological care in many countries, no direct access to a dermatologist in most countries, and several countries do not allow access to health care without any insurance certificate or cash payment. Comparing studies from different countries all over the world is difficult because of different and/or unclear, especially non-standardized definitions of itch, lacking to differentiate acute and chronic itch and especially of a lack of defining prevalence estimates. A very recent meta-analysis in atopic dermatitis (AD) and its clinical characteristics stated itch to be almost universally reported and the most common feature in AD (7). Chronic urticaria affects about 1% of the world population, presents with severe itch, and constitutes a global burden (8). Dermatological diseases (frequently accompanied by itch) have a worldwide distribution, but their prevalence is related to the geographical location. For example, otomycoses is much more frequent in subtropical and tropical climate (9) and does hardly contribute to the differential diagnoses of CI in countries with Western lifestyle. Scabies is considered to be the most frequent cause of acute itch but rather rare in CI. Scabies was recently added to the World Health Organization's list of neglected tropical diseases (NTDs), and its occurrence has been increasing in poor countries, countries with low standard of living, and especially in times of wars, migration, and in reception centers for asylum seekers (10). As infections and infestations are more prevalent in subtropical and tropical countries, scabies is a major cause of acute itch in these countries but is considered to be a less frequent cause in Western countries that are more affected by CI. All this may explain why itch is mentioned as a global burden of skin diseases, also in the elderly (11, 12). A large-scale implementation of a mass drug administration campaign of scabies in Ethiopia was called “stop the itch” (13).

Itch, especially CI, strongly reduces health-related quality of life (HRQOL) (2, 3, 5, 14–16). CI can be an additional burden in chronically ill patients leading to an additional reduction in QOL and affecting mortality in patients (14–16). CI can be a symptom or precursor of another (frequently) severe disease (17), but diagnostics are limited and not accessible in most countries; for example, radiological diagnostics and allergy testing are not reimbursed in many countries or excluded in insurance policy (18, 19). Common treatments like antihistamines do not relive itch, even in combination (19, 20). All this makes the topic of itch so important.

## Etiologies of Itch Worldwide: Examples

Itch is highly prevalent in European dermatological outpatient clinics. The prevalence of itch among adult dermatological patients was 54.4 and 8% among the controls (21). The intensity was highest among patients with prurigo. It is also prevalent in dermatological practices. In a German private practice, the prevalence of itch in a 1-week period was 36.2% (87.6% of whom had chronic itch) (22).

Itch may reflect a malignant disease. A Danish cohort study investigated the association between hospital inpatient and outpatient diagnosis of itch and cancer incidence (23). The 1-year absolute cancer risk was 1.63%. Among patients with itch, a 13% higher than expected number of cases with hematological and various solid cancers were found (23). However, the study was unable to differentiate between acute and chronic itch. In a Danish acute outpatient dermatological clinic, “pruritus and prurigo” were among the most prevalent diagnoses, accounting for 2.5% of all referred patients, but when considering all diagnoses, one may assume that itch was an accompanying symptom in more than 50% of referred cases (24).

Xerosis cutis, systemic diseases, and drug intake are frequent in elderly patients and may all contribute to the occurrence of CI (2, 3, 25). Dry skin, itchy dermatoses, and diabetes all contribute to chronic itch in the elderly.

Itch is also prevalent in the general population and is not always addressed by a medical doctor (MD). The Heidelberg Pruritus Prevalence Study showed a point prevalence of 13.5%, a 12-month prevalence of 16.4%, and a lifetime prevalence of 22% of CI in the general population (26). In the follow-up study, the 12-month cumulative incidence of chronic itch was 7%, and the lifetime prevalence was 25.5% (27). Women were more affected than men, and female sex was associated with an increased but non-significant risk for incident CI during the past 12-months (27).

Itch is prevalent in chronic diseases, especially in Western societies. CI in patients with end-stage renal disease (ESRD) is a considerable problem, and regional differences need to be taken into account because in (developing) countries, patients have limited access to hemodialysis (HD) with differing dialysis quality standards. There are no epidemiological studies on itch HD in developing countries because this treatment is hardly available. A representative cross-sectional prospective prevalence study on chronic itch (CI) in HD patients [German Epidemiological Hemodialysis Itch Study (GEHIS)] showed CI to affect 25.2% (point prevalence) of HD patients (28). No significant differences in prevalence estimates were shown concerning ethnic origin, schooling, or patients' marital status (28).

**Figure 1:**
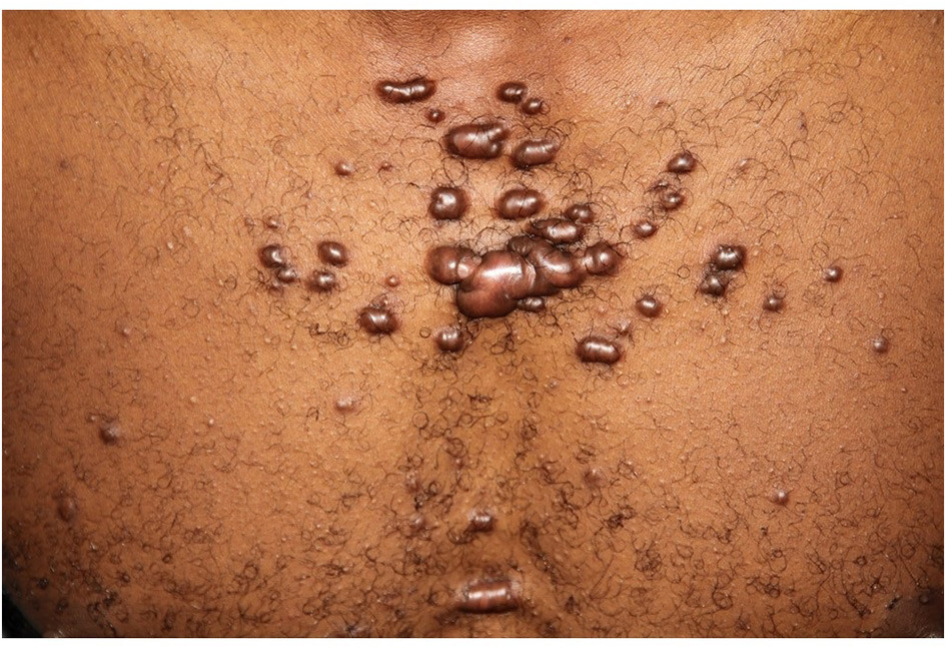
Ritual behavior like cutting the skin in many African and Asian countries contribute to chronic itch caused by keloid manifestation in variable shape and extent.

HIV infection and AIDS contribute to acute and CI worldwide (2, 3, 5, 29). Some diseases vary according to country and its most frequent diseases. Pruritic papular eruption (PPE) is a substantial cause of HIV-related morbidity in Sub-Saharan Africa. Its prevalence varies from 12 to 46% depending on the geographical region. More than half of HIV-infected patients report the eruption of PPE as their initial disease manifestation. We could show that none of the Ugandan itch patients had an underlying systemic disease, and in all HIV patients, itching was caused by dermatoses (5). It is likely that Ugandan patients with severe systemic diseases do not have a survival period that allows the initiation of itch, such as uremic itch, or access to certain therapies such as hemodialysis. Various etiologies from skin disease and keloids to drugs and burns contribute to a high prevalence of itch in African countries (30–34) (Figure 1).

## Discussion and Future Thoughts

Itch is a big challenge for clinicians and researchers all over the world, especially in regard to the structure of the regional healthcare system and the accessibility to medical care and specialists particularly in Non-Western countries. True epidemiological studies are lacking, especially in Asian and African countries as well as in South America. There is a lack of awareness, depending on, for example, the MD's speciality; other symptoms may be rated higher and/or itch is ignored. This is also caused by a lack of knowledge on itch. However, the rise in clinical and experimental research and findings resulting in an increased number of publications and networks like International Forum for the Study of Itch (IFSI) (1, 35, 36), the EDAV Task Force Pruritus (37, 38), and scientific meetings like The World Congress of Itch (WCI) have helped to increase itch being regarded as a global problem. Social networking platforms like, e.g., Instagram, connect patients looking for the support regarding itch (39). All this has increased the awareness of itch beyond dermatology. It has also improved the capacity to diagnose itch. More and specific clinical studies have enhanced the spectrum of treatments; local and systemic treatments were developed, which are also reflected in an updated S2k guideline on chronic pruritus (19). However, there is still a need for new and more specific treatments of itch including combination of treatments. The demographic situation in Western countries with an increasing number of aged patients, most of them chronically ill, will additionally increase the need for dermatological care including the treatment of itch. This also includes improving patients' motivation and the assistance to seek the specialist's help. Even if there is access, chronically ill patients tend to limit their visits to MDs especially when they already have chronic procedures/treatments like HD. This results in limited itch treatment, even in a Western country (40). In recent years, utilizing technology like telehealth services and teledermatology are useful to provide diagnoses like prurigo by consulting physicians (41). A global look makes one realize that all this is not accessible for most of the countries out of Europe and North America. The inequalities in health are also present in itch and its handling. There is a gross mismatch between the burden of itch and the skills and medications to treat them. To change this is basically not only a medical challenge but an economic and political one of all societies including the global economy. In the end, it is the task of any society to decide how to disseminate medical care and how to provide resources.

## Some Open Questions

How does the perception and the impact of itch differ according to continent and climate?

Will the climate change affect the frequency and etiologies of itch?

Does the awareness of itch and its health care depend on life expectancy and standard of living?

How can we improve education of MDs and healthcare providers to enhance the awareness for especially chronic itch?

Could we initiate a global network to better provide the care of chronic itch?

## Author Contributions

The author confirms being the sole contributor of this work and has approved it for publication.

## Conflict of Interest

The author declares that the research was conducted in the absence of any commercial or financial relationships that could be construed as a potential conflict of interest.
